# The Memory Effect of Reflected Self-Appraisals on Different Types of Others

**DOI:** 10.3389/fpsyg.2020.553585

**Published:** 2020-11-03

**Authors:** Caizhen Yue, Yajun Yang, Weijie He, Tong Yue, Weigang Pan

**Affiliations:** ^1^College of National Culture and Cognitive Science, Guizhou Minzu University, Guiyang, China; ^2^School of Psychology, Capital Normal University, Beijing, China; ^3^Laboratory of Emotion and Mental Health, Chongqing University of Arts and Sciences, Chongqing, China; ^4^Faculty of Psychology, Southwest University, Chongqing, China

**Keywords:** reflected self-appraisals, self-referencing, memory, remember-know (R-K) judgments, self-knowledge

## Abstract

The development of one’s self-concept unfolds within early interactions with intimate significant others for childhood and adolescence. Previous studies suggest that people define themselves in part through internalized perceptions of other people’s beliefs about them, known as reflected self-appraisals. Even in adulthood, reflected self-appraisals still remain critically influential on direct self-appraisals, and the affect might depend on the different types of others. In the present study, for the first time, we extend the classic “other-reference” paradigm to the field of reflected self-appraisals in order to examine whether there is a difference in the memory performance of reflected self-appraisals on different types of others in an early adult sample. In the experiment, participants were told to encode personality trait words by judging how different types of others (romantic partners, friends, and classmates) think about the participants themselves. After a retention interval, they received a surprise recognition memory test. The results showed that the memory performance of romantic partners is significantly better than that of friends and classmates, indicating that the memory performance of reflected self-appraisals varies across the others with different levels of closeness. Specifically, the closer the relationship between people and others is, the better the memory performance of reflected self-appraisals will be. Meanwhile, the speed and the encoding deepness of the reflected self-appraisals vary among different genders, leading to the gender effect of recognition memory. This study might help deepen our understanding on the development of self-concept in adulthood.

## Introduction

“Know yourself” is not just an old philosophical issue that arouses the interest of philosophers, such as Socrates, Plato, and Descartes (Vazire, 2010), but has also become a hot spot for psychologists (Cooley, 1902; Byrne and Shavelson, 1986). Self-knowledge is usually viewed as self-concept or an individual’s perception of his/her self. Cognitive psychologists propose that an individual’s view of the self is a “special” construct that engages unique organizational and elaborative processes (Gutchess et al., 2007). However, social psychologists emphasize the social component of the self and regard the self as a reflection of social life (Cooley, 1902; Mead, 1934). In general, self-knowledge is recognized as both a cognitive and social construction (James, 1890; Harter, 1999; Pfeifer and Peake, 2012).

How do we master the self-concept? The development of one’s self-concept unfolds within early interactions with caregivers and significant others (Murray et al., 2012). During adolescence, self-concept develops profoundly, self-evaluations become more complex, and peers and their opinions become increasingly salient (van Buuren et al., 2020). According to symbolic interactionism, one of the oldest but most influential theories of self-development, people define themselves in part through internalized perceptions of other people’s beliefs about them, known as reflected self-appraisals (Cooley, 1902; Mead, 1934). Pfeifer and Peake (2012) espoused that people’s self-views may be significantly shaped by the actual and/or perceived views of others (reflected self-appraisals). A number of studies have found the importance of reflected self-appraisals in the construction of one’s self-concept (Xu et al., 2015; Van der Cruijsen et al., 2019). In the process of internalization, reflected self-appraisals (what I think you think of me) were hypothesized to evolve into direct self-appraisals (what I think of myself) (Pfeifer and Peake, 2012). The process of reflected self-appraisals is validated in many fields, such as in the academic performance of middle school students (Bouchey and Harter, 2005; Tomasetto et al., 2015), a teacher’s teaching ability (Hu et al., 2014), professional athletic ability (Bois et al., 2005; Jose et al., 2015), juvenile delinquency (Walters, 2016; De Coster and Lutz, 2018), and the ethnic identity of ethnic minorities (Khanna, 2010; Sims, 2016). Several neuroimaging studies on the neural substrate of reflected self-appraisals also support this view (Pfeifer et al., 2009; Pfeifer and Peake, 2012; Romund et al., 2016).

Despite the importance of reflected self-appraisals in self-concept development, there is great discrepancy in the internal process. Specifically, some research found that reflected self-appraisals from different people have different effects on self-concept (Amorose, 2003). Previous research has noted that self-knowledge might be fully realized through the use of reflected self-appraisals from close others (Xu et al., 2015). This means that different types of others have different mental meaning for people. Accordingly, perceived opinions of others depend on the interpersonal closeness. In turn, reflected self-appraisals from distinct close others shape different self-concepts. The existing researches have found that information connected to the self allows for more efficient and deeper processing and encoding, which in turn facilitates later recall and recognition (Symons and Johnson, 1997). This is the well-known self-reference effect. Specifically, the memory benefits gained from self-reference have been found to extend to those with whom we are close (Mashek et al., 2003; Lee et al., 2016). When participants are asked to encode information in relation to a close other (other-reference), the overlaps in self and other representations are believed to facilitate memory processes (Serbun et al., 2011). This extension of the self-reference effect to close others may stem from a shared representation of self and close others (Ketay et al., 2018). Research from the cognitive, behavioral, and neural domains supports the idea of a shared representation between the self and close others (Aron et al., 1991; Gardner et al., 2002; Ketay et al., 2018). Taken together, both reflected self-appraisals and direct self-appraisals rely on the interpersonal closeness. Reflected self-appraisals can be viewed as a cycle of mutually influential judgments (Wallace and Tice, 2012).

The pattern of closeness for the self–other relationship varies across different cultures. The culture in which one is raised has a significant impact on the way one views himself or herself in relation to others (Triandis, 1995; Nisbett et al., 2001; Pfeifer et al., 2017). Markus and Kitayama (1991) distinguished the nature of the self from the cultural perspective, stating that people in Western cultures have independent selves, while Eastern cultures have dependent selves. Generally, East Asian cultures are characterized by thinking about things relationally (Gardner et al., 1999; Masuda and Nisbett, 2006). A cross-cultural study further demonstrated that Japanese college students’ self-concepts were more influenced by the presence of others than their American counterparts (Kanagawa et al., 2001). A study on the Chinese self-reference effect also found that the memory performances under the conditions of self-reference and mother-reference are equivalent (Zhu and Zhang, 2002). It is plausible that the development of self-concept may be affected by the unique characteristics of self–other relationship in different cultures.

In the process of self-concept development, the roles of reflected self-appraisals vary among different age groups. A lot of studies have found that a person’s self-appraisals in childhood and adolescence periods may be significantly shaped by his or her perceived views toward others (Cooley, 1902; Harter, 1999; Pfeifer et al., 2017). However, cross-cultural work suggests that reflected self-appraisals remain influential on direct self-appraisals in the process of development for members of collectivist cultures (Triandis, 1995; Gardner et al., 1999; Pfeifer et al., 2017). Therefore, it is necessary to study the characteristics of reflected self-appraisals in adulthood, especially those in the collectivist culture. The intimacy relationship is one of the most important social relationships in one’s early adulthood (Connolly et al., 2014). As Erikson’s eight-stage theory of life development states, the central issue in one’s early adulthood involves the establishment of intimacy (Erikson, 1985). Research on the process of reflected self-appraisals for Chinese young adults may help deepen our understanding on the development of self-concept.

The current study aimed to explore whether there are differences in the memory performance of reflected self-appraisals of different types of others among Chinese young people. Three types of others are selected – romantic partners, friends, and classmates – which show different levels of interpersonal closeness. On the basis of the previous researches, we raise a presumption that romantic partners are significant others for Chinese young adults. Moreover, reflected self-appraisals from romantic partners should have an important effect on their self-concept. Specifically, Chinese young adults may pay more attention to the opinions of their romantic partners and therefore encode these opinions deeply, which shows that romantic partners’ reflected self-appraisals display memory advantage. Moreover, previous studies suggested that women emphasize connectedness and sensitivity to others (Josephs et al., 1992), and they define themselves as higher in relational interdependence than men (Guimond et al., 2006). Accordingly, we speculate that males and females may have different memory advantages.

In order to study the memory process of reflected self-appraisals from different types of others, we conduct the revised remember/know self-reference paradigm in our experiment (Conway and Dewhurst, 1995; Ketay et al., 2018). In a classic self-reference task, at the encoding phase, participants were asked to rate personality traits with reference to themselves and to others. Following a retention interval, the participants were given an incidental recall or recognition test. The researchers believed that “remember” and “know” responses reflect qualitatively distinct components of recognition memory (Conway and Dewhurst, 1995; Carson et al., 2018). The “remember” responses reflect episodic memory, which in turn represents self-awareness. The “know” responses reflect the ability of semantic memory and represent the general awareness component (Conway and Dewhurst, 1995; Zhu and Zhang, 2002). Zhang et al. (2006) demonstrated that self-referential processing produced significantly higher proportions of “remember” and lower proportions of “know” judgments than do semantic-processing conditions. In the revised paradigm, the actual appraisals are replaced with reflected self-appraisals. The task of the experiment is to let the participants judge the trait words (do others think I am such a person?) and then conduct an incidental recognition test, which was a remember/know judgment. The revised paradigm was proven to be able to detect the memory advantage on the reflected self-appraisals for adolescence (Yue and Huang, 2012).

## Materials and Methods

### Participants

An earlier study exploring the differences between the two subject factors and three within-subject factors used a sample size of 56 (Tomova et al., 2019 with η_p_^2^ = 0.07). We relied on the effect size to estimate the required sample size in our study using the above eta-squared as input in G-Power 3.1 (Faul et al., 2007 with a power of 0.8 at an alpha of 0.05). Finally, it yielded a required sample size of 42, with at least 21 participants per group. Thus, we recruited 54 healthy undergraduates (27 males and 27 females, with a mean age of 20.24 years; SD = 1.06) to participate in our study.

All subjects were in romantic relationships (with a mean length of the romantic relationship of 18.81 months, SD = 15.86) and familiar with computer operations. All participants also had normal or corrected-to-normal visual acuity. Each subject received a written description of the study and provided informed and written consent prior to participation. This study was conducted in accordance with the recommendations of the Ethics Committee of the Chongqing University of Arts and Sciences. The protocol was approved by the Ethics Committee of the Chongqing University of Arts and Sciences. All subjects gave the written informed consent in accordance with the Declaration of Helsinki.

### Materials

A set of 240 Chinese adjectives were selected to compose a list of stimuli for encoding and recognition phases. The pleasure, meaningfulness, familiarity, and valence of the adjectives were considered and balanced based on the norms of Wang (2005). In all, 120 adjectives were presented at the encoding phase and the other 120 adjectives were used as lures at recognition. The study words were divided into six sub-lists (40 words each; 20 positive and 20 negative), matched on the basis of familiarity, meaningfulness, and pleasure from Wang’s (2005) norms (see Table 1). There were no significant differences on pleasure [*F*(5,239) = 0.072, *p* = 0.996], meaningfulness [*F*(5,239) = 0.115, *p* = 0.989], and familiarity [*F*(5,239) = 0.339, *p* = 0.889] across the six groups of adjectives. The number of characters in each sub-list of adjectives is equal (each adjective is composed of two to four Chinese characters). Each sub-list was assigned to one of three encoding conditions (romantic partners condition, close friends condition, or classmates condition) and counterbalanced across participants. Each sub-list was composed of half positive (e.g., generous and pleasant) and half negative (e.g., jealous and rude) traits. The order of presentation was randomized for each word for each participant, with trials from different conditions intermixed throughout the study.

**TABLE 1:** Descriptive statistical results of 240 Chinese adjectives of different dimensions.

	A	B	C	D	E	F
Pleasure	4.23 (1.41)	4.24 (1.66)	4.10 (1.58)	4.08 (1.63)	4.18 (1.57)	4.21 (1.61)
Meaningfulness	5.06 (0.19)	5.08 (0.22)	5.09 (0.23)	5.08 (0.19)	5.09 (0.18)	5.08 (0.20)
Familiarity	4.04 (0.49)	4.04 (0.48)	4.02 (0.49)	3.95 (0.51)	3.95 (0.50)	3.96 (0.52)

*Standard deviations are shown in parentheses. A–F, six sub-lists.*

The “Inclusion of Other in the Self” (IOS) scale (Aron et al., 1991) was used to measure the degree of interpersonal closeness. The IOS scale consists of seven pairs of overlapping circles, with each pair overlapping slightly more than the preceding pair. The participants were asked to select the pair of circles that best portrays their relationship with another (romantic partners, close friends, or classmates).

### Procedures

#### Questionnaire Task

After the participants entered the laboratory and sat down, they were asked to choose a close friend of the opposite sex, write down the name of that friend on the paper, and then describe the friend. The participants were next asked to choose a classmate of the opposite sex who was an acquaintance (rather than a close friend), write down that classmate’s name, and then describe the classmate. After the description was written down, the participants were asked to assess their levels of intimacy with lovers, friends, and classmates, respectively, using the IOS scale. After filling out the IOS scale, the participants were reminded that, in the subsequent experiment, when “friend” appeared on the screen, it referred to the friend whose name he/she had just written down. Likewise, when “classmate” appeared on the screen, it referred to the classmate whose name he/she had just written down.

#### Encoding Task

After receiving their instructions and practicing the adjective judgment task, the participants incidentally encoded adjectives in one of three ways: reflected self-appraisals of romantic partners (e.g., Does my romantic partner think that I am a friendly person?), reflected self-appraisals of friends (e.g., Does my friend think that I am a friendly person?), and reflected self-appraisals of classmates (e.g., Does my classmate think that I am a friendly person?). When “friend” (or classmate) appeared in the instruction, the experimenter reminded the participant to refer to the specific friend (or classmate) who was previously selected. Adjectives were presented on the computer screen for 4 s, during which time the participants pressed a key on a computer keyboard to provide a yes (S key) or no (K key) response. A total of 120 adjectives were encoded, with 40 adjectives assigned to each of the three conditions. Three counterbalanced orderings allowed the adjectives to be assigned to each condition across the participants, and trials were presented in a random order, with each trial unique to each participant.

#### Recognition Task

Immediately after the encoding task and a 6-min retention interval, the participants received instructions for the surprise recognition test, which was self-paced on the computer. The participants responded by pressing the F key to denote a previously studied word or the J key to denote a new word. If the participants pressed the F key, they indicated their subjective state of awareness accompanying that recognition by clicking on the “remember” (S key) or “know” (D key) option on the screen. The remember/know instructions were adapted from Gardiner’s (1988) study. The participants were instructed to select the “remember” option only if they were sure that they saw the word in the study phase and remembered some aspects of what was experienced at the time the word was presented. If they were sure that they had previously seen the word but could not remember any specific details about it, they were told to select the “know” option. Recognition was tested for all 120 encoded adjectives and 120 lures. Instructions placed equal emphasis on responding with accuracy and speed. Tasks were presented with E-Prime software.

## Results

### IOS Ratings

In order to compare the intimacy of the three types of others and the effect of gender, we conducted a 3 × 2 mixed analysis of variance (ANOVA) on the IOS ratings with encoding condition (romantic partners, friends vs. classmates) as the within-subject factor and gender (male vs. female) as the between-subject factor. The results are shown in Table 2. The ANOVA yielded a significant main effect of encoding condition [*F*(2, 104) = 167.195, *p* < 0.001, η^2^ = 0.763]. Specifically, the intimacy levels with romantic partners are significantly higher than those with friends and classmates (all *p* < 0.001), and the intimacy levels with friends are significantly higher than those with classmates (*p* < 0.001). The main effect of gender was not significant [*F*(1, 52) = 0.514, *p* = 0.477, η^2^ = 0.010]. The interaction effect of encoding condition × gender did not reach significance [*F*(2, 104) = 1.604, *p* = 0.206, η^2^ = 0.030].

**TABLE 2:** Descriptive statistical results of the Inclusion of Other in the Self (IOS) ratings of the encoding condition.

	Romantic partners	Friends	Classmates
Total	5.39 (1.16)	3.48 (0.97)	2.24 (0.75)
Males	5.63 (1.08)	3.44 (0.89)	2.22 (0.80)
Females	5.15 (1.20)	3.52 (1.05)	2.26 (0.71)

*Standard deviations are shown in parentheses.*

### Response Time in the Encoding Task

A 3 × 2 mixed ANOVA was conducted on the response time, with encoding condition (romantic partners, friends vs. classmates) as the within-subject factor and gender (male vs. female) as the between-subject factor. The results are shown in Table 3. The main effect of encoding condition was significant [*F*(2, 104) = 4.233, *p* = 0.017, η^2^ = 0.075]. The response times (RTs) with friends were significantly faster than with those of romantic partners (*p* = 0.041) and classmates (*p* = 0.013), while the RTs with the romantic partners and classmates were not significantly different (*p* > 0.05). The main effect of gender was also significant [*F*(1,52) = 5.711, *p* = 0.021, η^2^ = 0.099]. The RTs with the females were significantly faster than those of the males. The interaction effect of encoding condition × gender did not reach significance [*F*(2,104) = 0.114, *p* = 0.893, η^2^ = 0.002].

**TABLE 3:** Descriptive statistical results of response time (ms) of the encoding condition.

	Romantic partners	Friends	Classmates
Total	1,595 (434)	1,510 (389)	1,631 (421)
Males	1,703 (489)	1,638 (401)	1,744 (478)
Females	1,486 (346)	1,382 (336)	1,518 (326)

*Standard deviations are shown in parentheses.*

### Recognition Memory Performance

**FIGURE 1:**
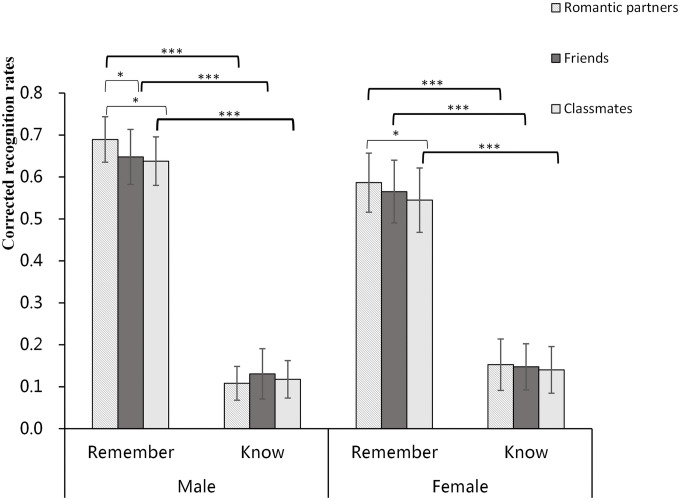
Corrected recognition rates of the different types of encoding condition. *Error bars* denote 95% CI. ^∗^*p* < 0.05; ^∗∗∗^*p* < 0.001.

A 3 × 2 × 2 mixed ANOVA was conducted on the corrected recognition scores, with encoding condition (romantic partners, friends vs. classmates) and response type (remember vs. know) as the within-subject factors and gender (male vs. female) as the between-subject factor. The results are shown in Table 4. The main effects of the three independent variables reached significance: encoding condition, *F*(2,104) = 3.624, *p* = 0.030, η^2^ = 0.065; response type, *F*(1,52) = 108.156, *p* < 0.001, η^2^ = 0.776; and gender, *F*(1,52) = 4.493, *p* = 0.039, η^2^ = 0.080. Specifically, the corrected recognition scores with romantic partners and friends were significantly better than those with classmates (all *p* < 0.05). Participants gave a significantly higher number of “remember” responses than “know” responses. And the corrected recognition scores with males were significantly better than those with females. The interaction effect of encoding condition × response type × gender was not significant [*F*(2,104) = 2.029, *p* = 0.143, η^2^ = 0.038]. The interaction effect of encoding condition × gender was not significant [*F*(2,104) = 0.753, *p* = 0.474, η^2^ = 0.014]. The interaction effect of response type × gender was also not significant [*F*(1,52) = 2.238, *p* = 0.133, η^2^ = 0.043]. However, the interaction effect of encoding condition × response type reached significance [*F*(2,104) = 4.043, *p* = 0.020, η^2^ = 0.072]. For the “remember” condition, the corrected recognition scores with romantic partners were significantly better than those with the friends (*p* = 0.037) and classmates (*p* = 0.018), but the difference between friends and classmates was not significant (*p* > 0.05). For the “know” condition, there was no significant difference among the three different encoding conditions (see Figure 1).

**TABLE 4:** Descriptive statistical results of gender differences in the correct recognition rate of the coding condition.

	Romantic partners		Friends		Classmates		Old		New	
	Male	Female	Male	Female	Male	Female	Male	Female	Male	Female
Total	0.798	0.723	0.785	0.714	0.757	0.681	0.779	0.706	0.352	0.275
Remember	0.689	0.586	0.648	0.565	0.638	0.545	0.658	0.563	0.231	0.184
Know	0.108	0.153	0.171	0.147	0.118	0.140	0.121	0.141	0.120	0.118

*Old: it appears in the existing adjectives. *New*: it does not appear in the existing adjectives.*

## Discussion

The present study is the first experiment to demonstrate memory performance on reflected self-appraisals of different types of others. For those in early adulthood, an intimate relationship is the most important interpersonal relationship (Erikson, 1985; Connolly et al., 2014). Therefore, we selected three types of others in the experiment: romantic partners, friends, and classmates. The task of the experiment was to ask the participants to judge whether others think that they are people with certain traits, and then an incidental recognition test was conducted. The results show that the memory performance of reflected self-appraisals varies with the degree of intimacy between individuals and others: the closer the relationship, the better the memory performance. Meanwhile, females encode information faster than males in the reflected self-appraisal process, but males gain better memory performance than females in the subsequent recognition task.

The present study recorded the response time during the encoding task. The results show that the response time under the friends condition was significantly faster than that for romantic partners and classmates. This result may indicate that the cognitive processing mechanism of reflected self-appraisals is different under the three types of conditions. A neuroimaging study revealed the internal encoding process and found that reflecting on a close other’s opinions about you (as compared to your own opinions of yourself) activated brain areas that are associated with the representation and regulation of emotional/motivational states. In contrast, reflecting a non-close other’s opinions about you activated areas that are involved in storing semantic and visual memories for people and objects (Ochsner et al., 2005). Due to the importance of intimacy in early adulthood, compared with the friends condition, the participants were apparently full of emotion and motivated when inferring romantic partners’ opinions about themselves. On the other hand, a lot of studies confirmed the overlaps in self and close others representations (Serbun et al., 2011; Ketay et al., 2018). It seems difficult to distinguish romantic partners’ opinions about themselves from their self-view. It may take up a lot of cognitive resources. Therefore, the reaction time was longer. In contrast, classmates are not relatively intimate and show a long psychological distance; therefore, participants have to rely on memories of prior experiences with them. Thus, it took a longer time to search for information in the memory to make judgments when inferring their classmates’ opinions about themselves. Further research is needed to demonstrate the internal cognitive processing of different types of others’ reflected self-appraisals. In this study, we found that the RTs with females were significantly faster than those with males. Bakan (1966) argued that a man is “egocentrist,” making himself the center of the world, while a woman is “alterocentrist,” making another person the center of her emotions. So a woman is considered as more sensitive to others (Josephs et al., 1992; Guimond et al., 2006). But a man needs more time to draw a distinction between his self-view and the opinions from close others, which involves a more deep encoding of information.

The present study finds that the proportions of “remember” are significantly higher than those of “know,” which is consistent with previous research (Zhu and Zhang, 2002; Zhang et al., 2006; Boduroglu et al., 2015). In self-reference research, researchers generally believe that remember/know is a very sensitive indicator of the self and that the remember responses reflect episodic memory, which in turn represents self-awareness (Tulving, 1985; Conway and Dewhurst, 1995). The know responses reflect the semantic memory and represent the general awareness component (Tulving, 1985; Zhu and Zhang, 2002). The results of the present study suggest that people usually have more self-processing when they infer others’ views on themselves.

In the present study, the memory performance of romantic partners is significantly better than that of friends and classmates on the remember condition. As mentioned above, the remember responses typically feature recollective experiences, whereby subjects can recall sensory aspects of the original events or thoughts and feelings that occurred during the event, while the know responses primarily feature feelings of knowing or familiarity (Conway and Dewhurst, 1995). When inferring a close other’s opinions about you, much emotion and motivation are involved in the process (Ochsner et al., 2005). As Symons and Johnson (1997) argued, the closer and more familiar a person is, the more organized and elaborate the information is about the individual. In early adulthood, one of the most important interpersonal relationships is intimacy (Zhou and Su, 2008; Connolly et al., 2014). Therefore, compared to friends and classmates, inferring romantic partners’ opinions can arouse more emotional details and episodic memory, which is conducive to deep processing of the information. Thus, the memory performance of romantic partners is better than that of others. It is worth noting that the corrected recognition scores for males are significantly better than those for females on the whole, which may be regarded as the by-product of a more cognitive resource allocation and a deep encoding of the opinions from close others for males (Bakan, 1966; Guimond et al., 2006).

It is interesting that our study found that people’s reflected self-appraisals of different types of others are hierarchical, which is in line with the hierarchy of other-reference (Symons and Johnson, 1997; Wang et al., 2019). That is, the closer the relationship between people and others is, the deeper people’s cognitive processing of how others perceive themselves will be and the better the memory performance of reflected self-appraisals will be. It is believed that the East Asian culture exhibit dependent selves (Markus and Kitayama, 1991), and one of the characteristics of the East Asian culture is interpersonal relationships (Yue and Huang, 2012). Thus, the memory effect of reflected self-appraisals on different types of others may be more significant in collectivist culture. In all, the results have verified our research hypothesis.

Previous studies on other-reference mostly make judgments on different types of others. The present study, for the first time, extends the other-reference to the field of reflected self-appraisals, which was to infer the views of different types of others on themselves. The study could promote the understanding of the cognitive processing mechanism of reflected self-appraisals. However, the study also has some limitations. The present study is a single experiment, and the sample is composed of Chinese college students, which makes the generalizability of the research limited. Therefore, future studies should include more diverse samples with respect to age and geographical region. For example, future research should investigate adolescents because previous studies have found that adolescent self-construal may rely more heavily on others’ perspectives about the self (Pfeifer et al., 2009). The present study focuses on romantic relationship. Besides romantic relationships, familiarity and importance are also important dimensions of interpersonal relationship. Future studies should examine diverse types of relationships in depth, such as kin relationships (e.g., siblings) (Platek and Kemp, 2009), familiar relationships (Mashek et al., 2003), and important relationships (Snodgrass et al., 1998). Finally, as for the content of reflected self-appraisals, the present study is only a general description and does not distinguish different domain-specific self-concepts. Previous studies have found that, in China, the social self is particularly interdependent, whereas the academic self is characterized by a relatively greater autonomy (Li, 2005; Li, 2006; Pfeifer et al., 2017). Therefore, future research should distinguish the content of reflected self-appraisals.

In conclusion, the present study finds the memory effect of reflected self-appraisals on different types of others; the closer the individual is to others, the better the memory performance will be. Females respond significantly faster in the encoding process than do males, but lose the memory advantage in the recognition memory task. People in early adulthood are more concerned about how romantic partners see themselves. The study shows that different types of other people’s reflected self-appraisals are hierarchical, which might promote our understanding of the cognitive mechanism of reflected self-appraisals.

## Data Availability Statement

The raw data supporting the conclusions of this article will be made available by the authors, without undue reservation.

## Ethics Statement

The studies involving human participants were reviewed and approved by the Ethics Committee of the Chongqing University of Arts and Sciences agreed to carry out the study, and all the procedures involved were in line with the sixth revision of the Helsinki Declaration. The patients/participants provided their written informed consent to participate in this study.

## Author Contributions

CY, WH, and TY designed the experiments. CY, WP, and YY carried out the experiments. CY and YY analyzed the sequencing data. CY and WP drafted the initial manuscript and revised the manuscript. All authors approved the final manuscript as submitted and agreed to be accountable for all aspects of the work.

## Conflict of Interest

The authors declare that the research was conducted in the absence of any commercial or financial relationships that could be construed as a potential conflict of interest.
